# Off-pump coronary artery bypass concomitant with retrieval of broken guide wire stuck in the brachial artery: a case report

**DOI:** 10.1186/s12872-021-01867-0

**Published:** 2021-01-22

**Authors:** Yuan Xue, Lu Dai, Wenjian Jiang, Hongjia Zhang

**Affiliations:** 1 Department of Cardiac Surgery, Beijing Anzhen Hospital, Capital Medical University, No. 2 Anzhen Street, Beijing, 100029 China; 2 Beijing Institute of Heart, Lung and Blood Vessel Diseases, Beijing, China; 3 Beijing Lab for Cardiovascular Precision Medicine, Beijing, China

**Keywords:** Off-pump coronary artery bypass, Retrieval of broken guide wire, Brachial artery, Coronary angiography

## Abstract

**Background:**

The broken guide wire could get stuck anywhere during coronary artery angiography, but the broken guide wire in the brachial artery is extremely rare.

**Case presentation:**

In this report, we describe our experience with a case of off-pump coronary artery bypass (OPCABG) concomitant with the retrieval of a broken guide wire stuck in the brachial artery: a 56-year-old male patient was referred to our hospital because of tri-vessel disease and a broken guide wire stuck in the right brachial artery. He received OPCABG concomitant with the retrieval of the broken guide wire stuck in the brachial artery under general anesthesia. The patient was discharged uneventfully, and 12 months follow-up showed an excellent surgical outcome.

**Conclusion:**

Open surgery is an effective means for treating patients with a guide wire stuck in the brachial artery during percutaneous coronary intervention.

## Background

The guide wire plays a key role in coronary angiography, and the broken guide wire stuck in the peripheral artery during angiography is rarely reported. The past 30 years has witnessed rapid development in the field of percutaneous intervention with gradually decreasing complications. However, there are still some complications that cannot be solved by the cardiologist, such as the guide wire stuck in the brachial artery. The broken guide wire in the artery may cause adverse outcomes including acute vessel occlusion, thrombosis, local or systemic embolization, perforation, and dissection [1]. In this report, we describe a case of off-pump coronary artery bypass grafting (OPCABG) concomitant with the retrieval of a broken guide wire stuck in the brachial artery.

## Case presentation

**Fig. 1:**
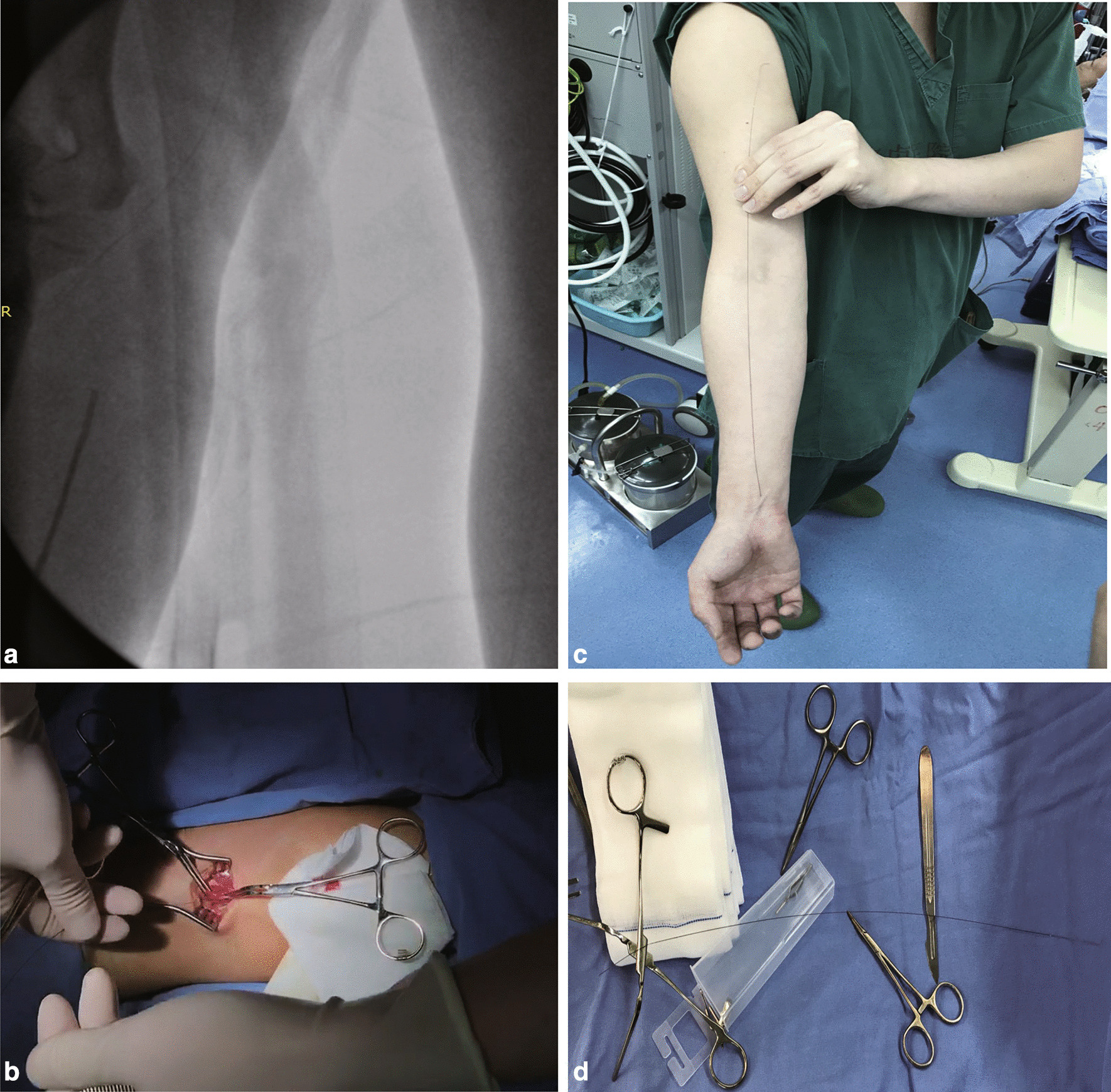
**a** X-ray showing the broken wire from distal of the radial artery to brachiocephalic artery. **b**–**d** The process of retrieve the broken guide from radial artery

A 56-year-old male patient received coronary angiography via the right radial route in a local hospital due to sudden onset of unstable angina pectoris. During the procedure, the cardiologist found that the right brachial artery was tortuous, and the guide wire was unable to pass through that region. Then, the cardiologist used the left radial artery to complete the angiography. The result showed tri-vessel disease suitable for coronary artery bypass grafting surgery. When they were removing the guide wire from the right radial artery, the guide wire broke and became stuck in the right brachial artery because of arterial spasm. They immediately infused heparin through the right radial artery to prevent thrombosis. Afterwards, they tried to pull the broken guide wire out through the femoral artery with a wire loop snare, but failed. The length of the broken wire was approximately from the distal radial artery to the brachiocephalic artery (Fig. 1a). The patient was referred to our hospital for emergency surgery.

The patient received OPCABG concomitant with the retrieval of the broken guide wire stuck in the brachial artery under general anesthesia. First, the right brachial artery was dissected and exposed. After clamping the proximal brachial artery, we incised it and removed the broken guide wire completely (Fig. 1b–d). Then the incision was closed as routine, and the emergency OPCABG was performed, including one arterial graft (left internal mammary artery to the left anterior descending artery) and one sequential great saphenous vein (aortic top end to the first diagonal branch to the obtuse margin branch to the posterior descending artery). The whole procedure was carried out smoothly, and no obvious guide wire residue was observed by postoperative chest X-ray. The patient was discharged uneventfully, and the 12 month follow-up showed an excellent surgical outcome.

## Discussion and conclusion

The broken guide wire could get stuck anywhere during coronary artery angiography, but the broken guide wire in the brachial artery is extremely rare [1]. The reasons the broken guide wire got stuck in the artery include excessive rotation, entrapment, and forceful traction [2, 3]. There is no consensus as to whether to remove the broken guide wire immediately and what kind of therapeutic measures should be used for this condition. Edward had reported a similar condition in a female patient [4]. Recent studies have shown that coronary artery angiography and percutaneous coronary intervention (PCI) are increasingly performed via the right radial route because of fewer vascular related complications [5]. However, the radial artery route is more difficult than the femoral artery route for the artery with a much smaller diameter, and it is more likely to spasm [6]. Flow-mediated dilation (FMD) value, radial artery diameter, number of PCI catheters exchanged and dosage of contrast media are the factors influencing brachial artery spasm during angiography and PCI [7]. Thus, measurement of the FMD before and after PCI, selection of the catheter approach, diameter of the catheter and use of antispasmodic drugs are useful in preventing vascular complications.

The consequences related to leaving in a residual guide wire include systemic or local thrombus, thrombosis, embolization, embolic phenomena, dissection, perforation, vessel occlusion and wire migration [8, 9]. These complications are life-threatening as they can lead to acute limb ischemia, infarction, or gangrene due to intravascular thrombosis. The potential approaches to this situation are percutaneous intervention, conservative management and surgical retrieval. The surgical retrieval will be the last choice if the percutaneous intervention therapy fails [2, 3]. When the broken guide wire was stuck, the cardiologist first tried to pull it out using the percutaneous method to cause less trauma. Commonly, percutaneous interventional therapy is mainly snaring the guide wire with a loop [3]. Since it could be performed immediately in the catheter lab with less invasive injury to patients than open surgery, this method is often considered as the first choice for the retrieval of the broken guide wire.

When the broken guide wire is stuck in the artery or the hemodynamic condition is unstable, surgery should be performed immediately. Additionally, excessive procedure in the vessels during percutaneous retrieval could potentially cause thrombosis, dissection, even rupture of the artery [10]. For these reasons and due to the patient’s tri-vessel disease, we chose the open surgery for this patient. In similar cases, it will be of great importance to consider the advantage of surgical retrieval and the concomitant revascularization. Once the broken guide wire is stuck, anticoagulant treatment should be started immediately in order to avoid artery thrombus formation, even if the patient is to receive surgical retrieval. In this report, two different methods were tried to retrieve the broken guide wire, including the percutaneous wire loop snare and surgical removal. This complication was finally solved by open surgery. Hence, both cardiologists and cardiac surgeons should work as a team to face this incident.

## Data Availability

Data sharing is not applicable to this article as no datasets were generated or analyzed during the current study.

## Abbreviations

OPCABG: Off-pump coronary artery bypass grafting
PCI: Percutaneous coronary intervention
FMD: Flow-mediated dilation

## References

[1] Datta G (2015). Broken guidewire—a tale of three cases. Indian Heart J.

[2] Tai S, Zhou SH, Tang L, Zhu ZW, Hu XQ, Fang ZF (2015). "Jailed-wire" technique is useful for percutaneous retrieval of entrapped guidewire. Int J Cardiol.

[3] Balbi M, Bezante GP, Brunelli C, Rollando D (2010). Guide wire fracture during percutaneous transluminal coronary angioplasty: possible causes and management. Interact Cardiovasc Thorac Surg.

[4] Danson EJ, Ward M (2015). Retrieval of a subintimal fractured guide wire from the brachial artery following saphenous vein graft stenting. Catheter Cardiovasc Interv.

[5] Hetherington SL, Adam Z, Morley R, De Belder MA, Hall JA, Muir DF (2009). Primary percutaneous coronary intervention for acute ST-segment elevation myocardial infarction-changing patterns of vascular access, radial versus femoral artery. Heart.

[6] Jolly SS, Amlani S, Hamon M, Yusuf S, Mehta SR (2009). Radial versus femoral access for coronary angiography or intervention and the impact on major bleeding and ischemic events: a systematic review and meta-analysis of randomized trials. Am Heart J.

[7] Heiss C, Balzer J, Hauffe T, Hamada S, Stegemann E, Koeppel T (2009). Vascular dysfunction of brachial artery after transradial access for coronary catheterization: impact of smoking and catheter changes. JACC Cardiovasc Interv.

[8] Lefèvre T, Louvard Y, Morice MC, Loubeyre C, Piéchaud JF, Dumas P (2010). Stenting of bifurcation lesions: a rational approach. J Interv Cardiol.

[9] Modi A, Zorinas A, Vohra HA, Kaarne M (2011). Delayed surgical retrieval of retained guidewire following percutaneous coronary intervention. J Card Surg.

[10] Almoghairi AM, Alamri HS (2013). Management of retained intervention guide-wire: a literature review. Curr Cardiol Rev.

